# Migrant Workers' Willingness to Participate in Workplace Health Promotion Programs: The Role of Interpersonal and Political Trust in China

**DOI:** 10.3389/fpubh.2020.00306

**Published:** 2020-07-14

**Authors:** Xinru Huang

**Affiliations:** School of Management, Xuzhou Medical University, Xuzhou, China

**Keywords:** workplace health promotion programs, interpersonal trust, political trust, subject cognition, health concern, political participation

## Abstract

High-level willingness to participate in WHPPs (Workplace Health Promotion Programs) can not only benefit employers and employees, but also can produce many positive social effects. In order to expand the existing body of research, the effects of subject cognition, interpersonal trust, political trust, and occupational safety and health concerns were explored. We surveyed 680 Chinese migrant workers who were in charge of participation decisions in their households (2,500 residents involved) from the three typical provinces. The association of social-economic determinants with the willingness to participate and the participating behavior was studied by logistic regression analysis. We find that almost all of workers show relatively high levels of willingness to participate, while nearly seventy percent of the migrant workers had not engaged in actual participation behavior. Regression analyses revealed that subject cognition, interpersonal trust, political trust, and concern for occupational safety and health were factors significantly influencing participating subjects' willingness to engage in WHPPs. Furthermore, mediation analyses demonstrated that the influence of subject cognition was partially mediated by political trust. The influence of subject cognition was partially mediated by political trust. We discuss why political trust may impact the influence of subject cognition on the willingness to participate. Our results provided important insights for both academic and practical application.

## Introduction

Migrant workers, who migrate from rural areas of their original residence to urban areas for work-seeking, are a unique group appearing in developing countries when experiencing economic transformation. Among these developing countries, China has the largest population, and wherein, has produced the largest human migration in history (1, 2). It was reported that there had been 168 million internal labor migrants (i.e., persons working outside of their home regions for at least 6 months) in 2015, more than 10% of its population (3). Furthermore, existing literature illustrated that In China, migrant workers have suffered the highest incidences of occupational diseases among various types of labor forces (4–6), mainly due to their exposure to poor working conditions (high concentration dust), occupational hazard (heavy metal pollution, occupational radiation) and long working hours (7, 8). In order to successfully manage occupational safety and health, strengthening citizen participation is an increasingly important aspect. In terms of occupational safety and social health governance, existing studies generally conducted meticulous and effective analyses from the perspective of the role and functions of government organizations, behavioral characteristics of participation subjects, and the occurrence mechanisms of social governance (9–11). By contrast, these studies paid less attention to the most fundamental subject: the participation of migrant workers in their own occupational safety and health governance. Most frequently, migrant workers were incorporated into the analyses as the targets of governance rather than the participating subjects of governance. As a result, there were few scholars placing emphasis on diversity of subjects (11, 12), the individual willingness and objective behaviors of migrant workers never obtained enough attention, and moreover, willingness of participation is integral to the success of occupational safety and social health programs. Therefore, OSH (Occupational Safety and Health) governance cannot achieve successful development without the participation of the migrant workers. The purpose of this research is to gain that understanding and explore the factors that influence migrant workers' decision to engage in OSH programs.

Workplace health promotion (WHP) is defined as preventing, minimizing and eliminating health hazards, and maintaining and promoting work ability. Wherein workers' health and wellness depends on various kinds of elements, such as physical and mental condition, social functions, healthy habits, energy and vitality. The healthy and productive workforce can bring economic benefits and citizen health (13). The concept WHP emerged as a popular strategy for improving health while providing cost benefits (14, 15). This type of program should remain prominent because of its ability to address workers' health rights and promote safe working environments (16). Workplace health promotion programs (WHPPs) aim to improve workers' overall health and well-being (17), including mental health, e.g., by reducing depression and anxiety (18, 19). These programs have provided positive work-related outcomes (14), such as reducing sickness-related absences (20), while increasing work productivity and attendance (13). Scholars have concluded that it is necessary to change health care spending from purchasing services for the passive treatment of occupational diseases to actively engaging in WHPPs (21). Various WHPPs have employed qualitative methods in their measurements (22, 23), analyzed the concept at organizational levels (17, 23), or emphasized the political framework (24, 25). Recently, Niessen et al. (26) analyzed whether individual characteristics and work-related determinants were associated with participation in a WHPPs. However, quantitative research regarding migrant workers' participation is relatively rare. There is a lack of research regarding individuals' attitudes toward WHPPs, and their willingness to participate in the programs. Consequently, research has not identified the factors that influence the decision to volunteer for WHPPs, or to invest time or money in such programs.

To address this gap in the research, our study investigated citizens' willingness to participate in WHPPs in terms of voluntary action and financial response behaviors. We focused on a subject's concern for occupational safety and health (OSH concern), cognitive consideration of the OSH governance participation (subject cognition), and interpersonal and political trust as the main factors that can influence willingness to participate in WHPPs. Specifically, social cognition models proposed that the participating behavior was derived from overall evaluation of the purpose, modes and effects of WHPPs (27–30). Self-cognition is also significant dimension, which can enable participants deal with confidence in individuals' knowledge and capabilities required to participate in WHPPs (31–33). Therefore, subject cognition should be considered to be an important factor influencing the willingness to participate in WHPPs. Additionally, in terms of trust, individuals with high levels of trusting tend to have more positive participation attitudes in political activities. Specifically, these people usually have more interest in communicating, cooperating and jointly participating in socially initiated activities (34). Except for these two factors, occupational safety and health concern is another significant element indicating how integrated one's interest in health is with one's daily activities, which also suggests an individual is health-oriented and has a positive attitude toward behaviors such as participating in WHPPs (27, 28, 35). We also considered the gap between actual participation behavior and behavior intentions (willingness to participate), because the concrete action is a step closer to civic engagement. Since the extent of the influence of these determinants on migrant workers' participation in WHPPs remains unknown, we proposed that a subject cognition, OSH concern, and interpersonal and political trust are the main factors influencing the willingness to participate. Understanding these determinants can provide better insights into the promotion of civic engagement in WHPPs. In turn, this research can highlight the steps needed to develop effective strategies to encourage migrant workers toward active participation and personal investment in WHPPs. In line with these goals, the current study addressed the following questions:

1. Are migrant workers willing to participate in WHPPs?
2. How does a subject cognition affect his/her willingness to participate in WHPPs?
3. How do interpersonal trust and political trust influence a subject's willingness to participate in WHPPs?
4. How do concerns about OSH influence a subject's willingness to participate in WHPPs?

To answer these questions, our study analyzed data from Chinese migrant workers, and employed multiple regression analyses and mediation analyses to examine the determinants of the willingness to participate in WHPPs. The remainder of this paper is structured as follows. Section Literature Review reviews the related literature and proposes the hypotheses. Section Data and Methods presents our data and methods, while Section Results provides our results. In Section Discussion, we discuss the results, and Section Conclusions offers our conclusions, with our comments about the limitations of this paper and considerations for future work.

## Literature Review

High-level willingness to participate in WHPPs can produce many positive effects in the field of public health promotion. Kim and Kim (36) found that it is necessary to develop an intervention program to improve college students' health. Richmond and McCracken (37) proposed that health promotion programs are worthwhile activities for elderly people. Employers, one of the benefits from participating WHPPs, is the improvement in morale and job satisfaction among employees (38). For employees, workplace health is considered vital method of health promotion, because the major people aged 18 to 64 would possibly spend a large amount of time there. Furthermore, such a healthier habitat at workplace can improve lifestyle in persons' daily life and the public health in the whole society. Therefore, in order to realize the aim to improve the participation in WHPPs, this research proposes the following hypotheses.

### Subject Cognition

Social cognition models can predict the health related behavior. One prominent model based on individual health cognitions is the planned behavior theory. In detail this theory proposed that behavior, for instance the participation in WHPPs, is mainly influenced be behavioral intentions (attitude, subjective norm, and perceived behavior control) (39). Specifically, attitudes arise of individuals' beliefs about the positive or negative consequences or an overall evaluation of the situation (40). This concept description of attitude can provide the theoretical foundation for sense of acceptance, which can be viewed as participants' overall evaluation of the procedures, modes and effects of WHPPs. Additionally, perceived behavior control is conceptually related to concept of self-efficacy, that deals with confidence in one's knowledge or skills required to accomplish a given task (e.g., participating to WHPPs). In the research of teachers' participating workplace health promotion program, Farokhzadian et al. (41) proved that the health promotion training program correlates positively with the self-efficacy for health practices. Therefore, participants' self-cognition has the positive effects on their participating decision in WHPPs. Except for this, within the scope of social governance participation, this field developed around two models: socioeconomic status and rational choice. Wherein the former model measures an individual's socioeconomic status by using factors including income, education level, occupation, and family background (31, 42), but it hardly ensures the universality and consistency due to social and cultural diversity, and further weakens the explanatory power of the socioeconomic status model (43).

The rational choice model supplements the socioeconomic status model to some extent. The rational choice model stresses that individuals have the motivation to participate in governance activities only as long as the gains resulting from individual political participation exceed the costs (44). Based on this, Chytil et al. (45) described human behavior toward health preservation, and further pointed that passive consumerism care should be excluded from the perspective of rational choice. This approach explains individual behavioral decision-making in participating to health promotion program to a large extent. Some research focused on various aspects of migrant workers' participation in WHPPs is also a sub-specialty within the study of citizen engagement in political participation (30). Verba et al. (42) proposed that political participation refers to those activities that can affect the decision making of the government personnel or the choices made by them. Riker (46) stressed that the concept of political participation has three defining constituents: citizens with meaningful opinions over government; these preferences are presented through a presentation system; and are considered to be fixed to democratic process. For example, Noehammer et al. (47) almost exclusively deals with decision making of the government personnel, when exploring determinants of the participation rates in WHPPs, and Linnan et al. (48) stressed citizens' direct and immediate involvement can enhance the power to decide local issues in workplace health participation. Thus, the point is to make citizens more involved in solving WHPPs, even if this take place within a larger framework of a representative political engagement activity.

However, both the socioeconomic status model and rational choice model have apparent shortcomings in their respective explanations of individual behavior. While the former explores the restriction on behavior imposed by individual socioeconomic status, and the latter observes the influence of individual rational considerations on behavior, both ignore the influence of social structure that can be described as the perception of individuals' own interactions, and the interactions between all others in multi-agent participation (49). Our research explored migrant workers' willingness to participate in WHPPs by combining individual subject cognition with the objective restrictions of social structure. According to the socialization theory, during the process when individuals transform from “living beings” into “social being,” i.e., during the process when individuals learn social norms and internalize social values, civic engagement can exert socialization effects. Such engagement enables participants to develop a more pro-social value pattern because of their interaction with like-minded others (50). Once individuals internalize social identification norms and values as part of their personal cognitive processing, they unconsciously conform to these norms and values in their behavioral practices. This essential part of their world view encourages them to participate in relevant social governance, thereby forming willingness to participate in social governance, with associated preferences in behavioral choices.

Our research rested on the following three perspectives used to measure the variable of subjective cognition. (a) Social governance participation: The willingness of migrant workers to participate, and the degree of actual participation, have an intimate connection with their personal opinions about rights and obligations. John (51) proved that citizens have several means to influence the public decisions, and similarly, policy makers can fine-tune their interventions to reach more groups of people. During this two-way interaction process, Nakayama (52) proposed that individuals could not only participate in a political decision-making process, but also show “participation action” after acquiring full, precise, and correct cognition about personal rights and obligations. (b) Sense of responsibility: For individuals, sense of responsibility constitutes self-monitoring of personal behavior. A sense of responsibility demands that the individual's actions meet the expectations of society under specific conditions. Behavioral game theory emphasizes that a sense of responsibility can stimulate individuals to make decisions by stimulating self-presentation motivation. A higher sense of responsibility and higher sense of self-monitoring will result more easily in individual behaviors that match up to social norms and expectations (53). For WHPP social governance, migrant workers who have a stronger sense of responsibility with respect to social governance are likely to play a positive part in relevant governance affairs. (c) Sense of acceptance for social governance: Society regulates goals, corresponding goal achievement, and institutional norms for every member of society as part of the entire cultural system. If individuals agree with such norms, and believe they can realize expected goals by complying with these norms, they are likely to adhere to the standards (54). From the perspective of migrant workers, the existing social governance model provides the norms that affect their social governance, identifying their rights and obligations to participate in social governance. In this sense, individuals who have a stronger sense of acceptance of social governance will increase the possibility of joining in social governance.

Hypothesis 1.1: Migrant workers with greater subject cognition have greater willingness to participate in WHP programs.

### Trust

Recently, a growing body of literature has been directed toward the concept of trust (55, 56), and this topic has been included in the research on political participation (29, 32, 33). In academic circles, there is a divergence of opinion about the conceptual definition and connotation of trust. One view regards trust as having certain expectations of others. Deutsch defined trust as “the expectation for a future time” and pointed out that such expectations would produce an impact on public decision-making behaviors (57). Sgro et al. (58) deemed trust to be certain psychological feelings whereby trustors are usually in the passive role and responsible for taking certain risks. Others viewed trust as a certain type of behavior. Messick and Kramer (59) indicated that trust refers to individual feedback behaviors based on judgments about the moral influences on others. Obviously, trust is a fundamental concept of interpersonal relationships and collaboration (60, 61). Luhmann (62) divided trust measurement into two dimensions: interpersonal trust and institutional trust. The former takes the affection among people as the bond that often exists in primary groups (such as families) and secondary groups (such as neighbors). Characterized by the intimacy of relationships, this bond leads to the intensity of trust. For instance, individuals have a stronger sense of trust in relatives than in neighbors (63). Political trust can be understood as citizens' confidence in political institutions, and this confidence can reflect their evaluations of the political environment (64). The decline in political trust can lead to a unique trend of political skepticism and civic disengagement that ultimately may affect the citizens' engagement in political activities (65).

There is little doubt that citizens who participate actively in political life display higher levels of political trust than passive citizens (66–68). This relationship between trust and civil engagement is complex. Trust demonstrates a positive correlation with volunteering activities (69) and financial investments in economic decision-making (70). Bäck and Christensen (71) found that generalized social trust is significant for the development for political participation, and argued that trust relates positively to citizens' propensity to become politically active. Dias and De Brito (23) emphasized the significance of trust for family health programs. Interpersonal trust can be considered as a kind of attitude toward others, or, perhaps, can best be conceptualized as how “trusting” an individual is (72). More trusting individuals will be more likely to commit themselves to community activities (e.g., health promotion program). It stands to reason that individuals with high levels of interpersonal trusting tend to have more positive participation attitudes in political activities. Specifically, these people usually have more interest in communicating, cooperating and jointly participating in socially initiated activities (34). However, the existing literature has neglected the analysis of different levels of interpersonal and political trust within the context of occupational health. The literature review above leads us to the following hypotheses.

Hypothesis 2: Interpersonal trust has a positive influence on the willingness to participate in WHPPs.

Hypothesis 3: Political trust has a positive influence on the willingness to participate in WHPPs.

In addition to the direct effect of trust on the willingness to participate in political projects, our research proposed the interaction of subjects' cognition with interpersonal and political trust. Trust exists in relationships with peers and especially with friends (73), and also with political institutions (74). Hence, migrant workers' beliefs about themselves play a significant role in whether they participate in social governance, such the governance of a community. Their subjective cognition may affect trust at the individual and collective levels. We expect that for individuals, higher levels of subject cognition positively correlate with higher levels of trust. For these reasons, we considered that subject cognition has an association with a workers' participation behavior through changes in trust, and we proposed the following hypotheses

Hypothesis 1.2: Interpersonal trust mediates the effect of subject cognition on willingness to participate in WHPPs.

Hypothesis 1.3: Political trust mediates the effect of subject cognition on willingness to participate in WHPPs.

### OSH Concern

It is of great significance to examine health cognition and perception in order to increase health promotion behaviors (75). Occupational safety and health concern is concept indicating how integrated one's interest in health is with one's daily activities, which also suggests an individual is health-oriented and has a positive attitude toward behaviors such as participating in WHPPs. Many researchers argued that workers' attitudes or concerns toward occupational safety and health can influence decision-making (27, 28). Lingard (35) argued that higher levels of concern about occupational health positively affect workplace health promotion behavior. Health-related attitudes have been found to be among the motivations for health promotion action (76, 77). Furthermore, Riley (78) demonstrated that occupational health awareness has a positive association with the willingness to support programs that promote workplace health. Therefore, we proposed the following hypothesis.

Hypothesis 4: OSH concerns have a positive effect on the willingness to participate in WHPPs.

### Conceptual Model

**Figure 1:**
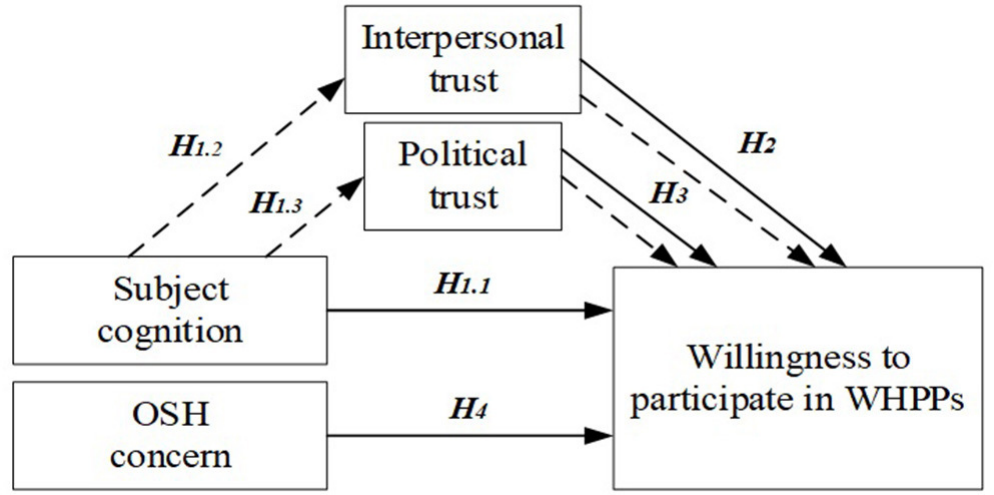
Conceptual model of the study.

Based on the hypotheses, our study proposed a conceptual model that can test and examine determinants influencing the willingness to participate in WHPPs. This model emphasizes the analysis of specific factors derived from the relevant literature, and contains three main components: subject cognition, OSH concerns, and trust (interpersonal and political trust) in Figure 1. In addition, the model also includes social-demographic determinants, because the empirical evidence demonstrated that such factors have an effect on civic engagement in political activities (79, 80). Literature review and identification of the real problems were the basis for addressing research hypotheses and for building the conceptual model, listed below.

- The subject cognition has s a direct, positive and statistically significant effect on the willingness to participate in WHPPs (H1.1).
- Interpersonal trust mediates the effect of subject cognition on willingness to participate in WHPPs (H1.2).
- Political trust mediates the effect of subject cognition on willingness to participate in WHPPs (H1.3).
- There is a direct, positive and statistically significant relationship between the interpersonal trust and willingness to participate in WHPPs (H2).
- There is a direct, positive and statistically significant relationship between the political trust and willingness to participate in WHPPs (H3).
- There is a direct, positive and statistically significant relationship between the OSH concern and willingness to participate in WHPPs (H4).

To summarize, the theoretical model for the determinants of the willingness to participate in WHPPs integrates determinants from the theory of planned behavior, rational choice and the concept of political participation from political theories (39, 40, 81). The benefit of this model is that it identifies the important factors relevant in evaluating the subject cognition and OSH concern and their relationships with the willingness to participate in WHPPs. Additionally, the model assists researchers in determining what constituent parts might be relevant in the study of a particular phenomenon. This aligns with our intention of proposing a conceptual model which aids the researcher in the study of workplace health promotion and sustainable development.

## Data and Methods

### Data

To determine whether interpersonal trust and institutional trust have an impact on migrant workers' willingness to participate in workplace health promotion programs, we used field investigation data gathered by research groups in Anhui, Henan, Shanxi, and Shanxi Provinces. The research sample involved more than 2,500 residents in ~750 households. Using a random sampling approach, the investigation selected 750 rural households from Huaibei City in Anhui Province, Xuzhou City in Jiangsu Province, Xi'an City in Shanxi Province, and Taiyuan City in Shanxi Province. The researchers chose one family member who was familiar with family production and operation conditions as the respondent. Field investigation work in Huaibei, Taiyuan, and Xuzhou City proceeded from March through May 2017, while field work in Xi'an City took place in November 2017. There were 680 questionnaires altogether. It is worth mentioning here that for obtaining more precise investigation results, members in the research group donated small gifts worth about 20 RMB to every respondent prior to beginning formal questioning.

### Model Selection

The main goal of our work was to explore the influence of trust on migrant workers' willingness to participate in WHP programs. Therefore, the explained variable of the research is migrant workers' participation willingness for WHP programs. This variable is defined as discrete (0-1) to avoid reflection problems in identification. In a linear model, personal characteristics would affect the explained variable in a linear manner (82) and accordingly lead to reflection problems. As demonstrated by the research of Brock and Durlauf (83), in Probit, Logit, and other non-linear models, reflection problems can be avoided. According to model fitting effects, in our research we selected the binary logistic model on the individual level to analyze the key influential factors of migrant workers' willingness to participate in WHPPs.

Suppose migrant workers' participation willingness is determined by the following formula:

(1) $$\begin{array}{l} \text{log }it\;(willingness_{i}=1)=\;\Phi \;(\alpha_{i}\;control\;variables_{i}\;+\;\beta_{i}\;trust_{i}\\ \;+\;x_{i}\;cognition_{i}\;+\;\partial_{i}\;concern_{i}), \end{array}$$

Wherein the subscript i means the investigated. The explained variable “willingness” is the 0-1 variable about peasant participation willingness for WHP programs. If migrant workers are willing to participate in WHP programs, the value should be 1, or otherwise 0.

### Measures

The face-to-face interviews included questions designed to reveal the subjects' interpersonal trust and political trust, subject cognition, and OSH concerns (Table 1). The main constructs were measured as follows.

**Table 1:** Sample characteristics.

**Variables**	**Sample (*****N*** **=** **680)**		**Variables**	**Sample (*****N*** **=** **680)**	
	**Absolute**	**(%)**		**Absolute**	**(%)**
Gender			Number of migratory cities		
Female	169	24.853	1	139	20.381
Male	511	75.147	2	188	27.647
Age	3	189	27.794		
18–29 years	67	0.099	4	82	12.059
30–39 years	322	47.353	5	38	5.589
40–49 years	265	38.971	6	30	4.412
50–59 years	25	3.676	7	14	2.059
60 years and older	1	10.000			
Highest professional qualification	Employment sector				
No professional qualification	64	9.412	Metallurgy & mining industry	267	39.265
Primary school	226	33.235	Construction	155	22.794
Secondary school	209	30.735	Manufacturing	95	13.971
Certificate from a specialized technical colleges	139	20.441	Hotels/restaurants	82	12.023
Qualification from a university and above	42	6.176	Domestic service	43	6.323
Monthly income (RMB: yuan)	Wholesale/retail	32	4.706		
<1500	38	5.572	Entertainment	6	0.882
1501–2500	143	21.029			
2501–3000	213	31.232			
3001–3500	227	33.382			
>3500	59	8.676			

#### Attitudes Toward OSH

The research measured migrant workers' attitudes toward OSH based on workplace health promotion. For example: (a). I would like to be offered a workplace program that promotes health. (b) I think that I would benefit from these OSH projects. Responses were assessed on a Likert-type scale from 1 (strongly disagree) to 5 (strongly agree) based on Röttger et al. (40). Other questions included workplace context, for example: If an OSH project was initiated by the government in your work place, with the goal of promoting migrant workers' workplace health status, in general, what would be your attitude toward this project? Responses to questions regarding attitude toward an OSH project promoting workplace health were measured on a Likert-type scale ranging from 1 (very negative) to 5 (very positive).

#### Willingness to Participate

We used three questions to measure migrant workers' willingness to participate in OSH projects. Are you willing to volunteer for an OSH project with the objective of calling attention to occupational disease groups and raising awareness of occupational hazards prevention? [In general, how high is your willingness to invest time in, or volunteer for, an OSH project (84)?] Are you willing to invest financial resources in an OSH project? (In general, how high is your willingness to invest money and contribute financially to an OSH project?) Each of these two items was rated using a Likert-type scale ranging from 1 (very low) to 5 (very high). The two items were significantly correlated with each other (Spearman's rho = 0.801). Therefore, we used the average score for these two items to represent willingness to participate in this research.

#### Subject Cognition

For this research, we chose three variables to reflect migrant workers' subjective cognition: cognition of rights and obligation, cognition of responsibility, and acceptance of social governance. For evaluation, we used the scale employed by Zhang and Li (85). The three response items were assessed using a 5-point, Likert-type scale from 1 (strongly disagree) to 5 (strongly agree). The three items formed an internally consistent scale (Cronbach's alpha = 0.809). For example, For example, (a) The governance of relevant major affairs of occupational safety and health requires the participation of migrant workers; (b) Participating in OSH governance is the responsibility and obligation of the self and other migrant workers; (c) You are very satisfied with present OSH governance modes.

#### Trust

Based on data from the European Social Survey (42) regarding the general measurement of trust, we further specified the objects that can be trusted. With the combination of social norms defined by Fugas et al. (86), we adjusted these five trust scale, extending it further to include peer influence on the willingness to participate in OSH projects. Hence, the concept of trust studied in our research was not trust in a general sense. Instead, we looked at migrant workers' actual rational behavioral expectations or effective identity with others up to personal interests in OSH governance. In our investigative process, we considered migrant workers' comprehension ability and acceptance, and transformed the five types of trust above into the following five research questions: (a) “I trust in relatives very much; (b) “I trust in friends very much; (c) “I trust in the government very much; (d) “I trust in OSH policies and regulations very much.”

#### OSH Concerns

To measure the degree of OSH concerns, we employed the scale applied by Lingard (35). It contained three question items, such as the following. (1) I think there is a high likelihood of experiencing a work-related injury or illness in my current occupation. (2) I think my work is very dangerous. (3) I think the measures taken by OSH to control risks are sufficient (enough). These items were measured on a 5-point, Likert-type scale ranging from 1 (strongly disagree) to 5 (strongly agree). The mean of the items (Cronbach's alpha = 0.829) was employed to represent the OSH concerns.

#### Control Variables

Regarding the selection of control variables (Table 2), “individual” is the individual characteristic vector that affects migrant workers' participation willingness. As proved by existing studies, gender, age, and education level all have significant impact on the participation rates in WHPPs (79–81). Consequently, personal characteristic variables chosen in our research included gender, age, education level, Number of migratory cities (the number of cities which workers migrate to in search of work), and employment sector. “Household” is the family characteristic vector that affects participation willingness, including net household income. The influence of these variables on WHPPs participation willingness has been proven (87). Therefore, family characteristic variable selected for this research included is family monthly income (measured by the monthly income in the family from a single source).

**Table 2:** Scale, internal consistencies and items for measurement.

**Scale**	**Cronbach's α**	**Items**	**Mean (*SD*)**
Willingness to participate	0.801	1. In general, how high is your willingness to invest time in or volunteer for WHPPst? (time)	4.303 (0.450)
		2. In general, how high is your willingness to invest money and contribute financially to WHPPs? (money)	3.901 (0.576)
Subject cognition	0.809	1. The governance of relevant major affairs of occupational safety and health requires the participation of migrant workers.	4.519 (0.280)
		2. Participating in OSH governance is the responsibility and obligation of the self and other migrant workers.	3.619 (0.281)
		3. You are very satisfied with present OSH governance modes.	1.720 (0.285)
Trust	Interpersonal trust (0.829)	1. I trust in relatives very much: (family)	3.785 (0.762)
		2. I trust in friends very much: (friends)	3.753 (0.854)
		3. I trust in neighbors very much. (neighbors)	3.549 (0.991)
	Political trust (0.853)	1. I trust in the government very much. (government)	2.557 (0.776)
		2. I trust in OSH policies and regulations very much. (policy)	1.169 (1.105)
OSH concern	0.829	1. I think it is high likelihood of experiencing a work-related injury/disease in my current occupation?	3.407 (0.794)
		2. I think my work is very dangerous.	3.238 (0.690)
		3. I think the OSH risks controlling measures are sufficient. (enough)	2.735 (0.703)

## Results

To test our hypotheses, binary logistic regression and multiple regression analyses and mediation analyses were performed using SPSS Statistics (version. 23). First, we tested hypotheses H1.1, H2, and H3, represented as continuous lines in the conceptual model. In the next step, we tested the mediation effect of H1.2 and H1.3 using PROCESS software, employing a practice used in prior research (88).

### Descriptive Statistics

**Figure 2:**
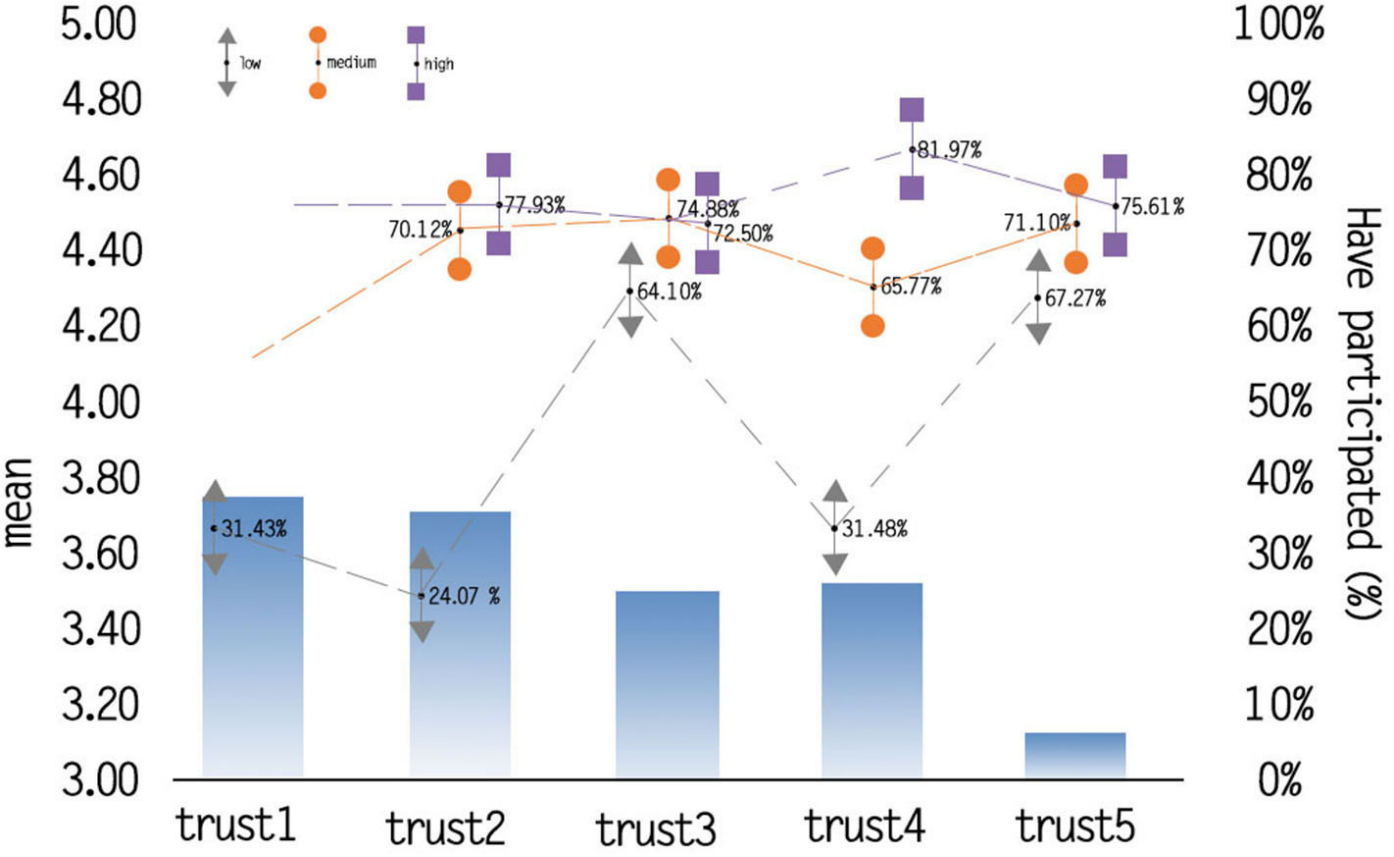
Means and the participating percentages among the dimensions of trust.

As is reported in Figure 2, the means of political trust (trust 4 and trust 5) is far below than interpersonal trust (trust 1, trust 2, trust 3). Specifically, the means of trust 1 (the trust to family) is highest (mean = 3.785, *SD*= 0.762; 5-point scale), followed by trust 2 (the trust to friends) (mean = 3.753, *SD*= 0.855; 5-point scale), while the means of trust 5 (the trust to policies) is lowest (mean = 1.169, *SD*= 1.105; 5-point scale). Specifically, each trust dimension was divided into low, medium, and high categories, obviously showing that the percentage of individuals, who had participated in WHPPs, represents an upward trend with the increasing degree of trust. A point should be noted that the percentage of actual participating behavior is similar among medium and high profiles of respondents.

According to Table 3, all the determinants in our study are found to be positively and significantly related to willingness to participate. There is a positive correlation between political trust and subject cognition, OSH concern and interpersonal trust. On the contrary, we found a positive correlation between interpersonal trust and subject cognition, political trust and interpersonal trust, OSH concern and subject cognition. Hence, except for the relationship between OSH concern and political trust, we find significant associations between subject cognition, trust and OSH concern.

**Table 3:** Correlation matrix.

**Spearman's rho**	**Subject**	**Interpersonal**	**Political**	**OSH**
	**cognition**	**trust**	**trust**	**concern**
Subject cognition	1			
Interpersonal trust	−0.305^***^	1		
Political trust	0.406^***^	−0.136^***^	1	
OSH concern	−0.900^***^	0.221^***^	−0.003	1
Willingness to participate	0.242^***^	0.305^***^	0.879^***^	0.096^**^

** p < 0.01,

*** *p < 0.001 (2-tailed)*.

Table 4 summarizes the all the variables' descriptive statistics used in our conceptual model. As is shown that the mean of subject cognition (mean = 4.021; *SD*= 0.280; 5-point scale) is highest among exploratory variables, followed by OSH concern (mean = 3.955; *SD*= 0.378; 5-point scale), and interpersonal trust (mean = 3.702, *SD*= 0.562; 5-point scale). Each participants showed relatively high willingness to participate, but there is still exceed 70 percent of Chinese migrant workers failing to participate in WHPPs.

**Table 4:** Descriptive statistics of determinants used in the analysis.

**Measures (*N* = 680)**	**Maximal**	**Minimal**	**Mean**	**SD**	**Std**.
	**value**	**value**			**error**
Subject cognition	1	5	4.021	0.280	0.011
Interpersonal trust	1	5	3.696	0.562	0.022
Political trust	1	5	1.863	0.676	0.026
OSH concern	1	4	3.955	0.378	0.014
Willingness to participate	1	5	4.250	0.225	0.009
Participate or not (%)	Yes	28.382	No	71.618	

### Regression Analyses

As is shown in Table 5, the model including all variables explains a substantial proportion of the dependent variable according to Adjusted R square, increasing from 0.005 to 0.211. The analysis presents that subject cognition (B = 0.281, *p* < 0.001), OSH concern (B = 0.161, *p* < 0.001), interpersonal trust (B = 0.180, *p* < 0.001), political trust (B = 0.147, *p* < 0.001), and gender (B = −0.072, *p* < 0.001) significantly correlate with the willingness to participate in WHPPs. Hence, the aforementioned results offer support for H1.1, H2, H3, H4. A point should be noted, females is found to be higher participation rates than males, which means that gender has predictive value for willingness to participate. Overall, subject cognition are found to have the highest effect on willingness to participate in WHPPs, followed by interpersonal trust, OSH concern, and political trust.

**Table 5:** Parameter estimates for the regression analyses.

	**Unstandardized**		**Standarduzed**	**Unstandardized**		**Standarduzed**	**Unstandardized**		**Standarduzed**
	**coefficients**		**coefficients**	**coefficients**		**coefficients**	**coefficients**		**coefficients**
	**B**	**Std. error**	**Beta**	**B**	**Std. error**	**Beta**	**B**	**Std. error**	**Beta**
Subject cognition				0.193^***^	0.019	0.362	0.150^***^	0.023	0.281
OSH concern				0.083^***^	0.015	0.201	0.067^***^	0.015	0.161
Interpersonal trust							0.050^***^	0.010	0.180
Political trust							0.034^***^	0.010	0.147
Gender	−0.011	0.013	−0.034	−0.023^**^	0.011	−0.072	−0.023^**^	0.011	−0.072
Age	0.000	0.001	−0.018	0.000	0.001	−0.018	0.000	0.001	−0.020
Highest professional qualification	−0.013	0.014	−0.087	−0.001	0.013	−0.009	0.001	0.012	0.009
Employment sector	0.002	0.014	0.005	0.001	0.013	0.004	−0.002	0.012	−0.007
Number of migratory cities	0.001	0.005	0.011	0.000	0.005	0.002	−4.493	0.005	0.000
Net household income	−0.015	0.013	−0.100	−0.003	0.012	−0.023	0.001	0.012	0.009
(Constant)	4.129^***^	0.091		2.928^***^	.127		2.928^***^	0.127	
	*Adjusted R square −0.005*			*Adjusted R square 0.171*			*Adjusted R square 0.211*		

** p < 0.01,

*** *p < 0.001. Dependent variable: willingness to participate in WHPPs*.

### Mediation Analyses

In order to test whether the influence of subject cognition is mediated be changes in interpersonal trust and political trust. We tested the mediation through using PROCESS macro, which is similar to Preacher and Hayes (89)'s research. The bootstrapping teats reported in Table 6 shows that the indirect effect of subject cognition on willingness to engage in WHPPs through interpersonal trust is non-significant (indirect effect = −0.0006, 95% CI = [−0.0115, 0.0103], on the precondition that the socio-demographic determinants are controlled. On the contrary, the indirect effect through political trust is positive and significant (indirect effect = 0.0464, 95% CI = [0.0255, 0.0773]. The direct effect was found to be positive and significant (indirect effect = 0.1509, 95% CI = [0.1064, 0.1954]) when mediators had been included in the model. Therefore, as we hypothesized, subject cognition affects willingness to participate in WHPPs, and this effect can be influenced through changes of political trust rather than interpersonal trust. That is, H1.3 are supported. The results reveals that political trust can partially mediate the relationship between subject cognition and willingness to participate.

**Table 6:** Results of mediation analysis with PROCESS.

**Exogenous variable (X)**	**Mediator (M)**	**Endogenous variable (Y)**	**Coeff. B**	**Coeff.b**	**Effect (a × b)**	**BC bootstrap 95% CI**	
			**B (X → M, a)**	**(M → Y, b)**			
Subject cognition	Interpersonal trust	Willingness to participate	−0.0093	0.0603^***^	−0.0006 (indirect)	−0.0115	0.0103
Subject cognition	Political trust	Willingness to participate	1.3420^***^	0.0346^***^	0.0464 (indirect)	0.0255	0.0773
Subject cognition		Willingness to participate			0.1509 (direct)	0.1064	0.1954

*** *p < 0.001; control variables were environmental concern, age, gender, net household income. The indirect effect of subject cognition on willingness to participate through trust as simultaneous mediators is presented*.

## Discussion

Our findings revealed that all migrant workers showed relatively high levels of willingness to participate in WHPPs, but almost 70 percent of the respondents failed to engage in actual participation behavior. This finding was in line with prior opinions (90) that a gap exists between behavior intention and actual behavior. Pai and Edington (90) proposed that behavior intention (i.e., willingness to participate in programs) can produce a positive effect on conducting actual behavior. Hence, our work further demonstrated that various types of barriers prevent participants from engaging in political activities. Our study proposed that these barriers can be identified as the subject cognition, OSH concern, and trust. With regards to subject cognition, although workers believe that, as an indispensable part of social governance, they have responsibility and obligation to take part in WHPPs, they showed rather unsatisfied with current OSH governance modes. That is, current governance mode is hardly acceptable for workers, which could act as a barrier for them to engage in political activities. On the one hand, migrant workers, as the governed object, could possibly feel less confidence in their knowledge, capacity required in participating to WHPPs, and further accomplishing the goal of health promotion. The main reason could be explained that they are socially less desirable group with lower socioeconomic status (91) and lower educational level (92). In terms of trust, the means of political trust was far lower than for interpersonal trust. Specifically, among the dimensions of trust, the individual's trust of family was the highest form of interpersonal trust, but the trust of OSH policies was lowest. Compared with political trust, interpersonal trust was often understood as a “cultural feature” that is closer to the degree of an individuals' general trust (93). This can be explained that individuals who have a greater propensity toward trusting other people are more likely to show higher levels of interpersonal trust in terms of different personalities (94). Our study showed that migrant workers with higher levels of interpersonal trust while relatively lower levels of political trust, in particular lowest trust in OSH policies. This finding may serve as an alert that policy makers and related institutions should pay more attention to the citizens' attitudes toward the government and its policies.

Among sonic-demographic variables, gender, monthly household income, and educational background have predictive value for willingness to participate to WHPPs, which is also examined in the research on the participation rates of African Americans recruited to a health promotion program (95). Specifically, females were found to have higher participation rates than males. These findings were in accordance with the research on gender differences in participation rates by Bernstein (96), who argued that women workers are more politically interested and active than their male counterparts. These findings highlighted the need to move toward a revised view of women engaging in political activities (97). Feminist research on women workers' higher participation rates proved empirically that females are significantly more politically active than men. For example, in terms of private activism, females are more likely to sign petitions, and boycott or buy products for political reasons. This private individualistic behavior is incorporated into their political participation more easily (97–99). Migrant workers' with lower household incomes or lower educational levels were more likely to have experience participating, which is in line with the findings of Cicatiello et al. (100)'s research. In that work, Cicatiello et al. argued that political involvement is positively affected by income, mainly because political activities are costly and require the investment of personal resources (e.g., money, skills). Our study held the opinion that migrant workers tend to work in urban areas to increase their income. Hence, they have more knowledge of occupational safety and health, and are more likely to participate in WHPPs. In addition, individuals with lower educational levels are persuaded easily to participate in collective activities, and therefore, they are more likely to participate in WHPPs. This result is similar to the findings of prior research indicating a link between and political participation (101–103). These scholars argued that education offers citizens the skills and resources needed to participate in political activities.

Therefore, in order to better design the program for promoting migrant worker's health, some insights can be gained from the results as follows: (1) the program is relatively feasible from theory significance, but the additional effort required is very high. The external policy advocacy and additional political education lessons are necessary. (2) The government and the relevant governing institutions should actively deliver the policy-oriented values to citizens and effectively enable migrant workers know the benefits of various policies. At the same time, it is necessary to re-establish and strengthen the trust bond between the migrant workers and the government, as well as the perception of the existence, obligation and utility perceived by them in the participating process of political activities. (3) Another important point is the investigation of participants' knowledge, acceptance and satisfaction toward programs, which is part of the program design. The participants should be divided according to the characteristics of population variables (e.g., education, income level). Based on this, the government should formulate targeted policies with regard to different groups, aiming to maximize the willingness to participate in WHPPs.

## Conclusions

This study aimed to provide a better understanding of civic engagement in political activities by studying Chinese migrant workers' willingness to participate in WHPPs. We proposed and tested hypotheses based on a conceptual model that focused on subject cognition, interpersonal and political trust, and OSH concerns. All the interviewed Chinese migrant workers had the intention to engage in WHPPs. However, almost seventy percent of the participants did not actually participate in WHPPs, as measured in terms of their workplace health promotion behavior. Our analyses revealed that subject cognition, interpersonal trust, political trust, and OSH concerns were positively associated with the willingness to participate. Subject cognition and OSH concern were the most strongly positively associated with the willingness to participate, followed by interpersonal trust, and political trust. In our study, gender was the only one of the social-demographic variables that could predict the willingness to participate in WHPPs. Interpersonal and political trust demonstrated a partial mediation effect through subject cognition on the willingness to participate. From the viewpoint of all dimensions, male migrant workers' participation rates did not correlate significantly with their trust in their neighbors (trust 3). Likewise, the participation rates of migrant workers with a high-education level did not demonstrate significant correlation with trust in families (trust 2), neighbors (trust 3), or policies (trust 5). However, interpersonal and political trust, and their five dimensions, showed significant correlation with gender, income, and education levels.

This paper highlighted the need for research regarding interpersonal and political trust as measured in terms of workplace health promotion. In so doing, we aimed to contribute to the academic research on workplace health promotion and related political participation. Our study can enlarge the scope of social-psychological research into workers' intentions and behavior regarding programs that promote workplace health. Certain individual characteristics, such as trust, can be used as indicators to determine willingness to participate. Furthermore, the gap between intention and behavior is also addressed in our study. The data regarding actual behavior can advance the relevant research on civic engagement in WHPPs. Furthermore, our findings can help improve the initiation and smooth operation of WHPPs through consideration of the effects of trust, subject cognition, and OSH concern. This approach can improve related policy planning and increase citizens' responses. The high levels of willingness to participate in our study revealed that the policy makers should establish an appropriate management framework that could help migrant workers to form sustained participation in WHPPs. Meanwhile, related OSH policies should pay more attention to lowering the costs and barriers for workers' participation rates in WHPPs, with the aim of improving OSH management. Specifically, raising health-care consciousness at workplaces may offer a foundation for active participation. Consciousness-raising can be accomplished through OSH-related education and training programs aimed at informing employees of the individual personal benefits they can achieve. In this way, an increasing rate of civic engagement in WHPPs in China could improve the management of the national occupational safety and health scheme.

## Data Availability Statement

All datasets generated for this study are included in the article/supplementary material.

## Ethics Statement

The studies involving human participants were reviewed and approved by China University of Mining and Technology. The patients/participants provided their written informed consent to participate in this study.

## Author Contributions

XH: Conceptualization, Data Collection, Formal analysis, Writing-original draft, and Writing- review and editing.

## Conflict of Interest

The author declares that the research was conducted in the absence of any commercial or financial relationships that could be construed as a potential conflict of interest.
